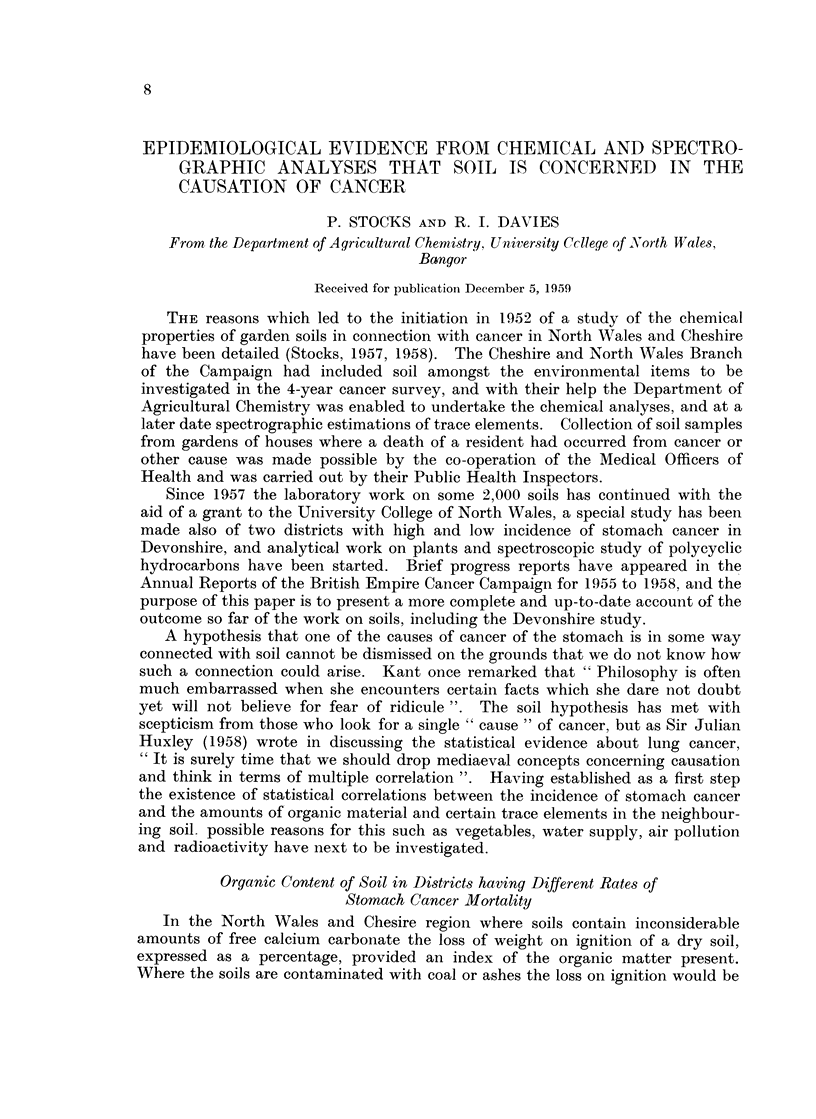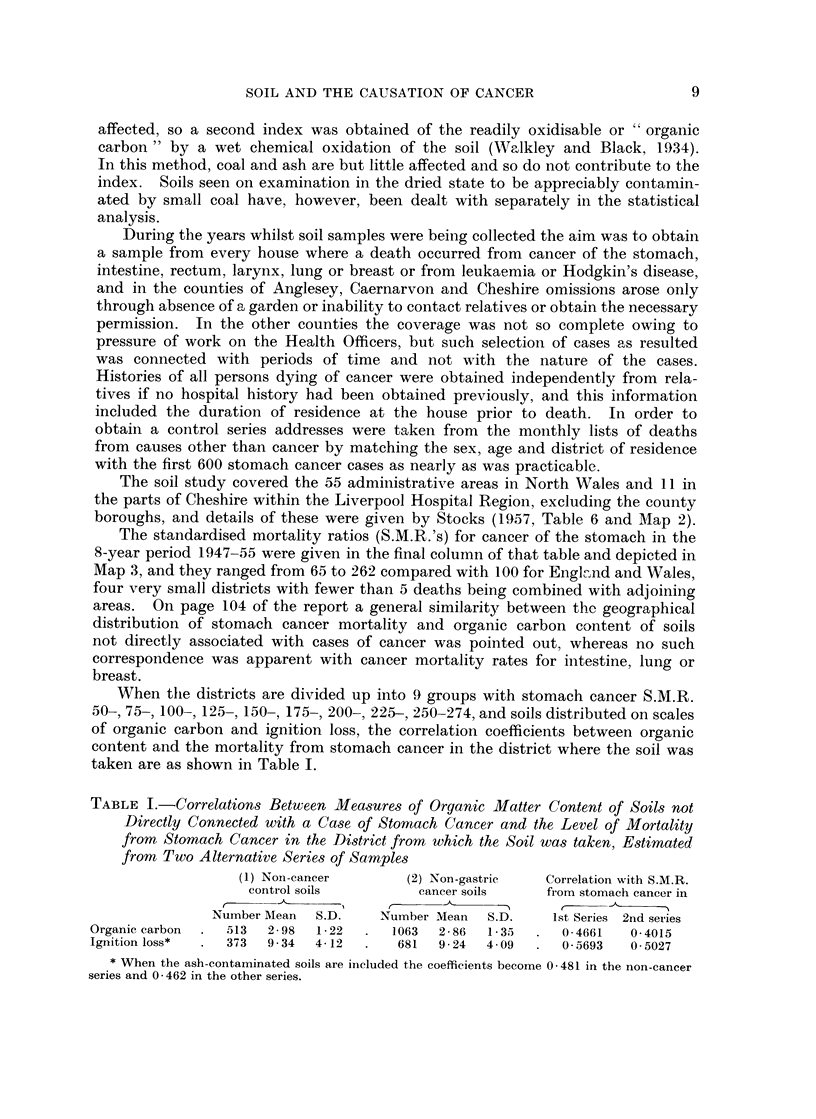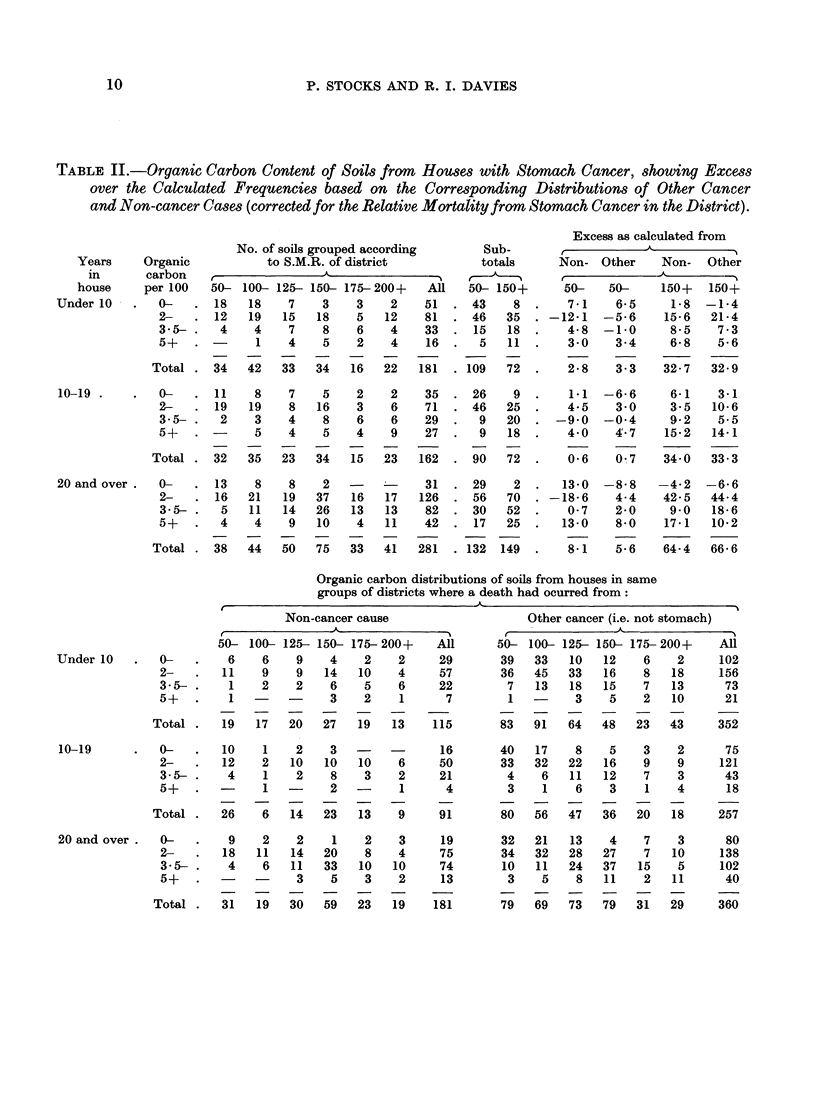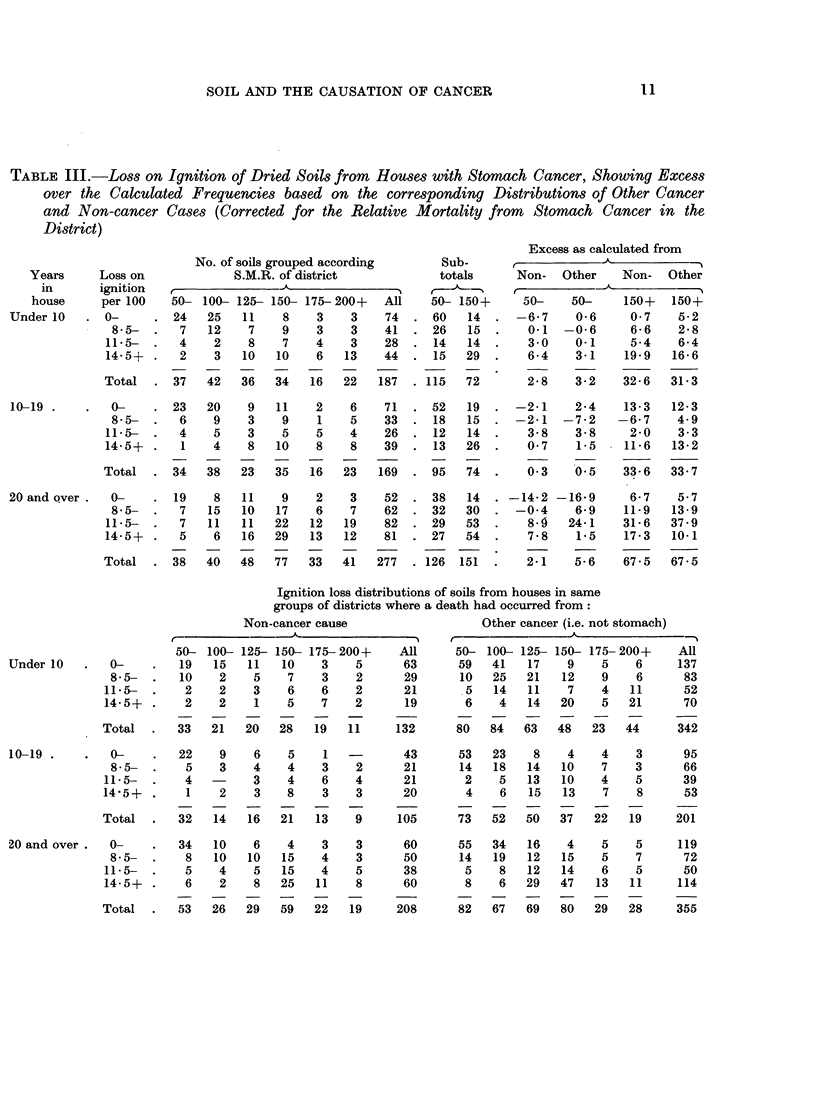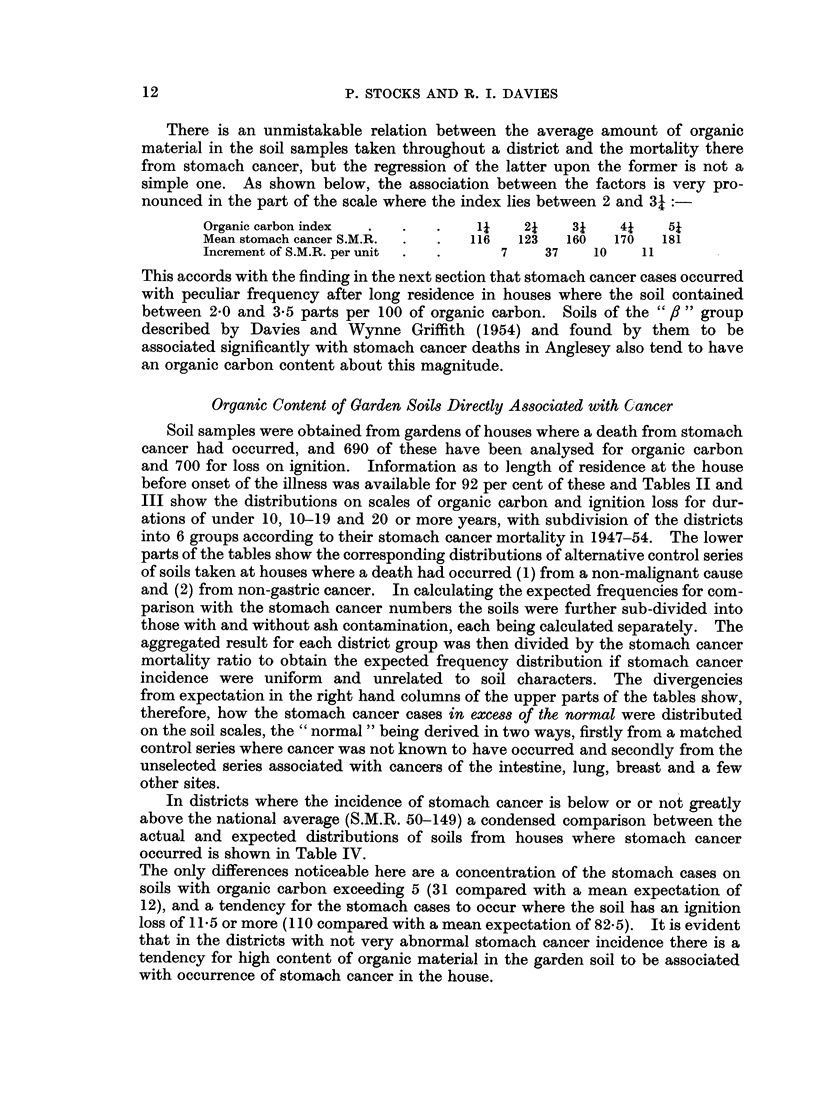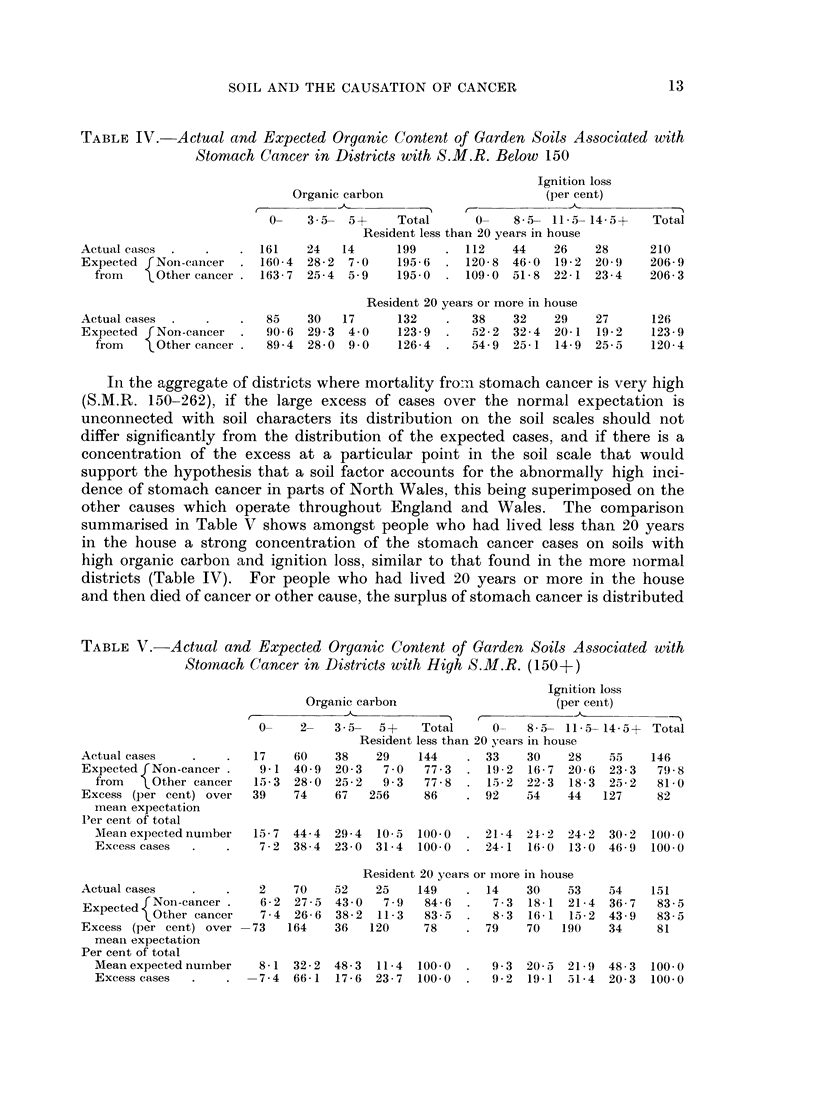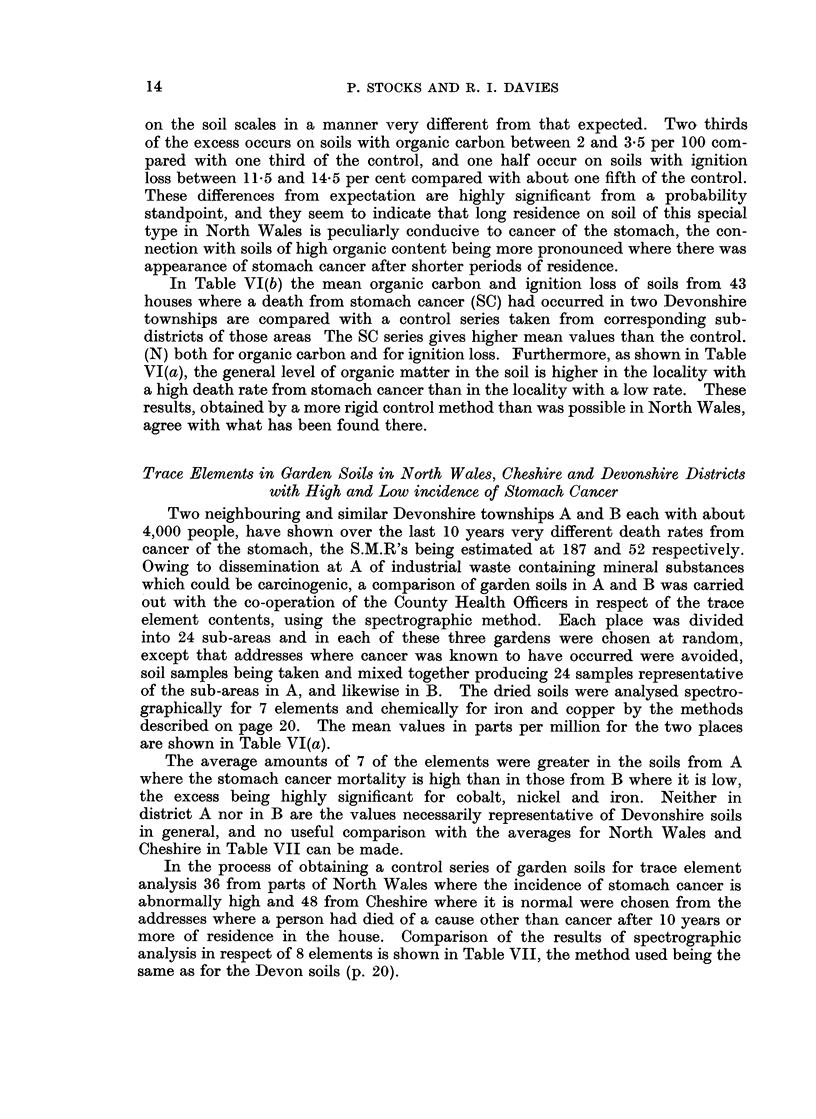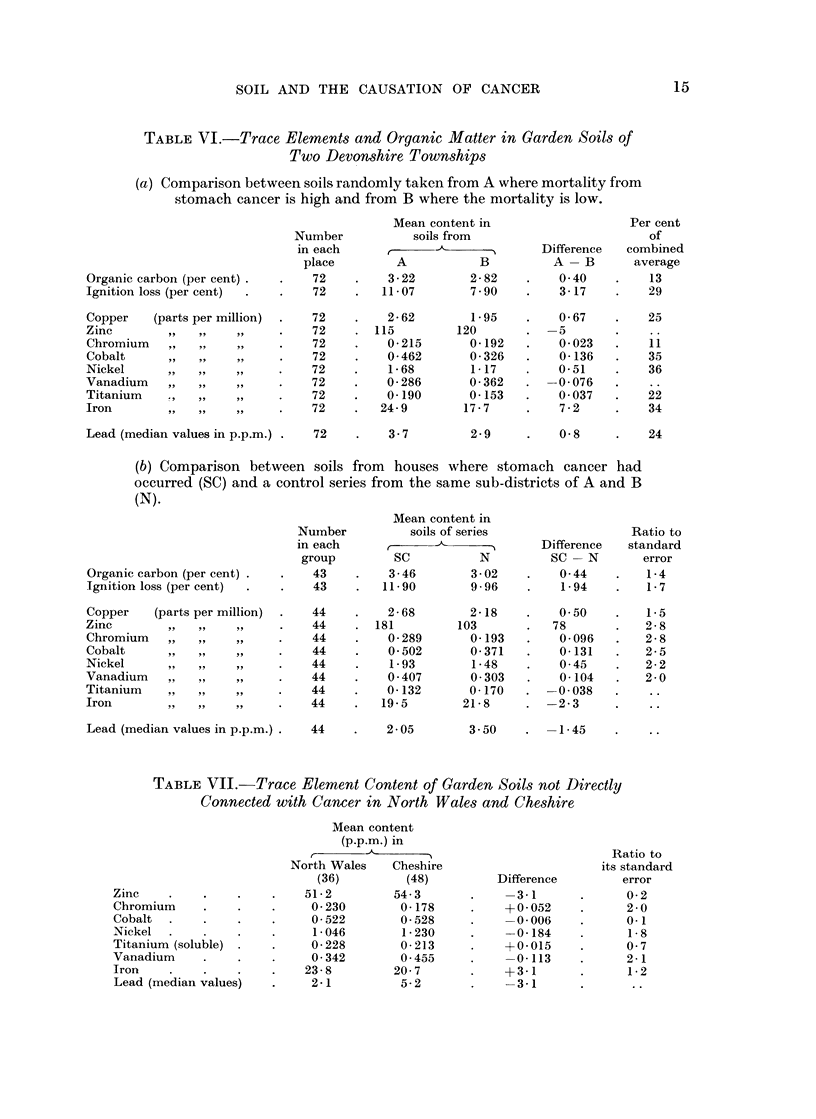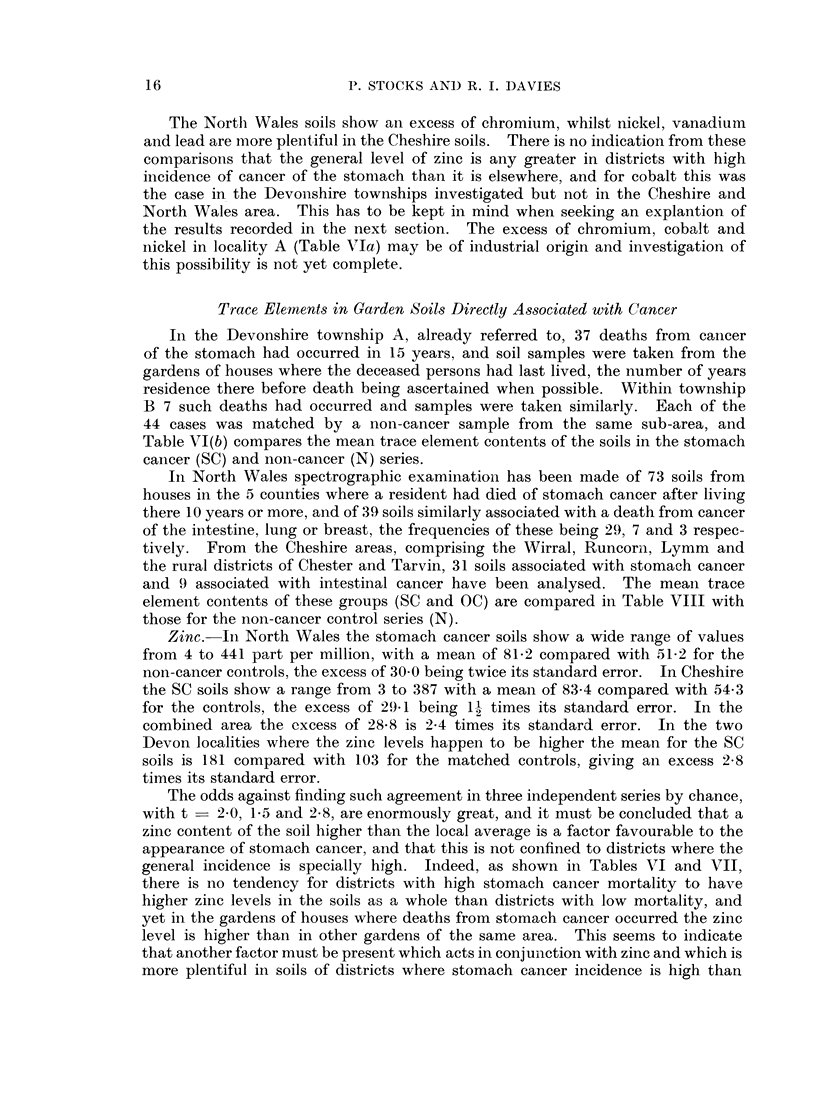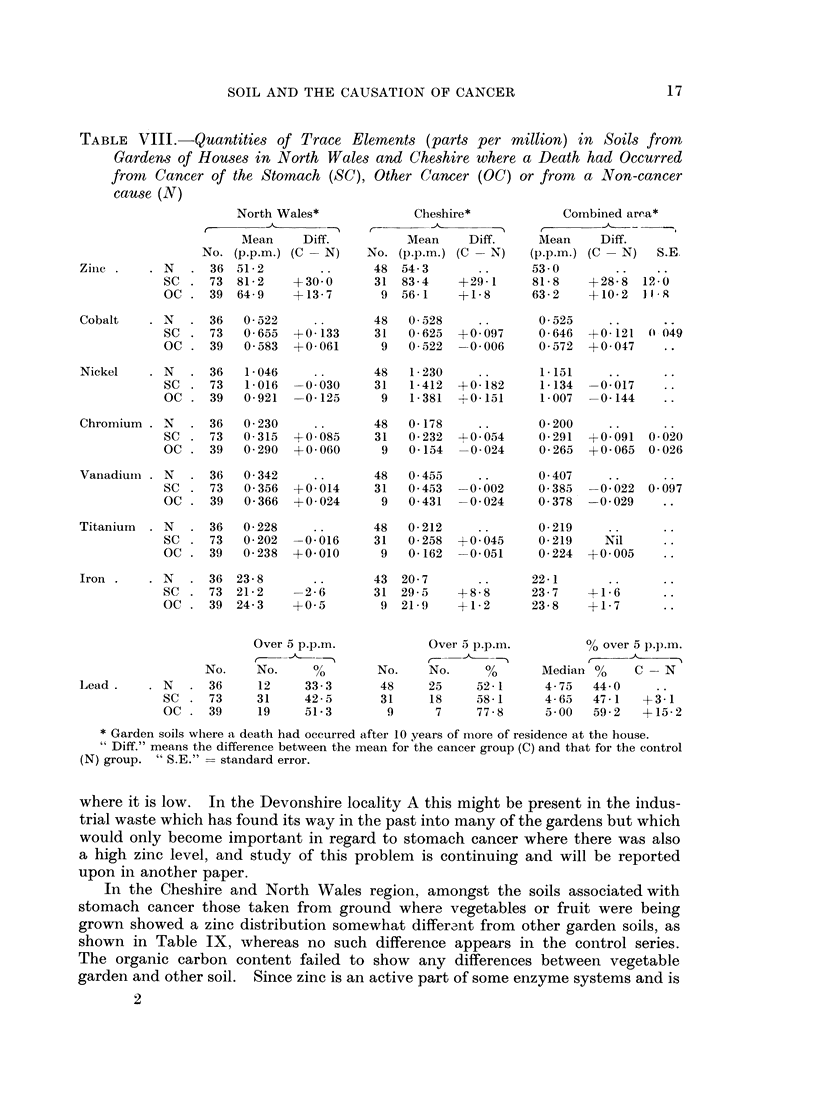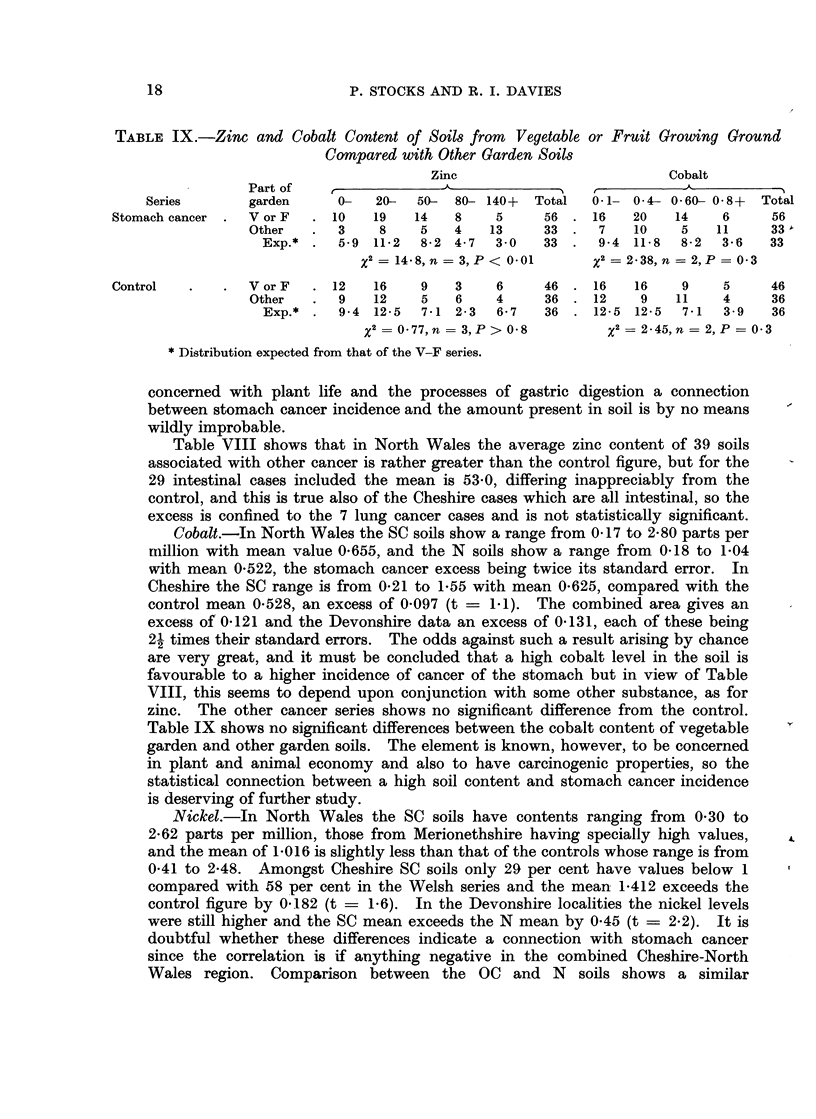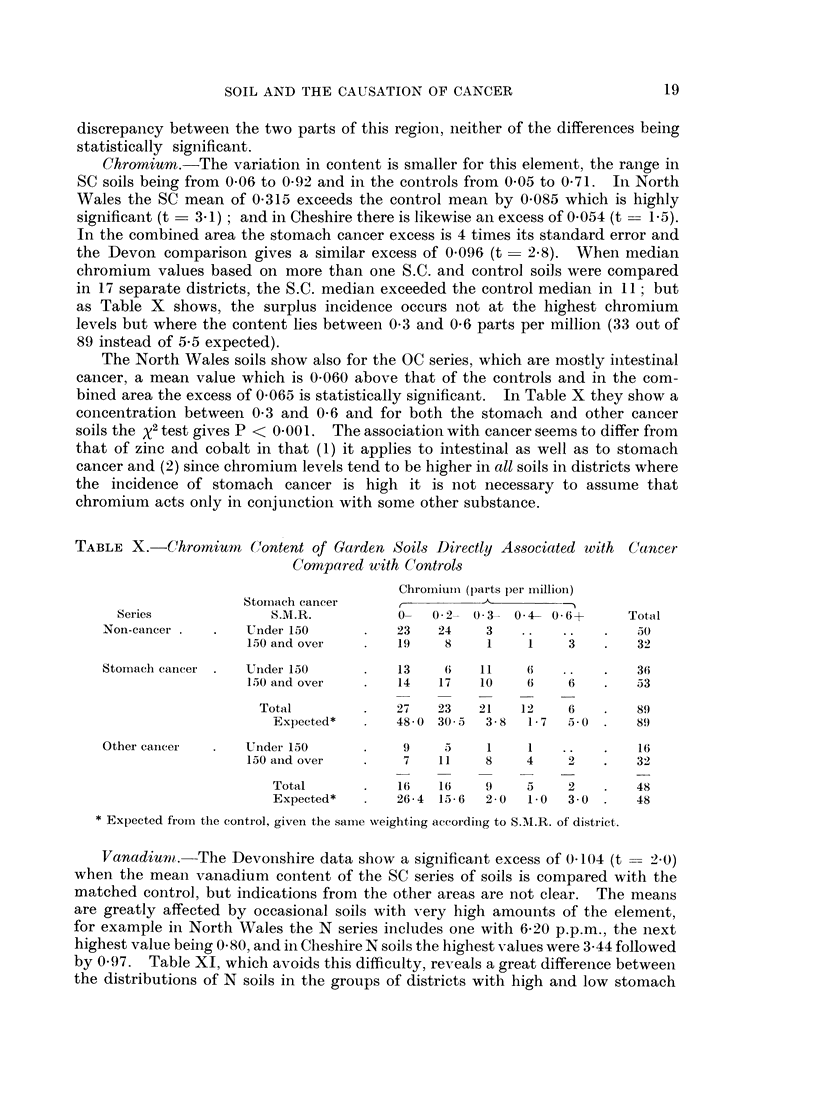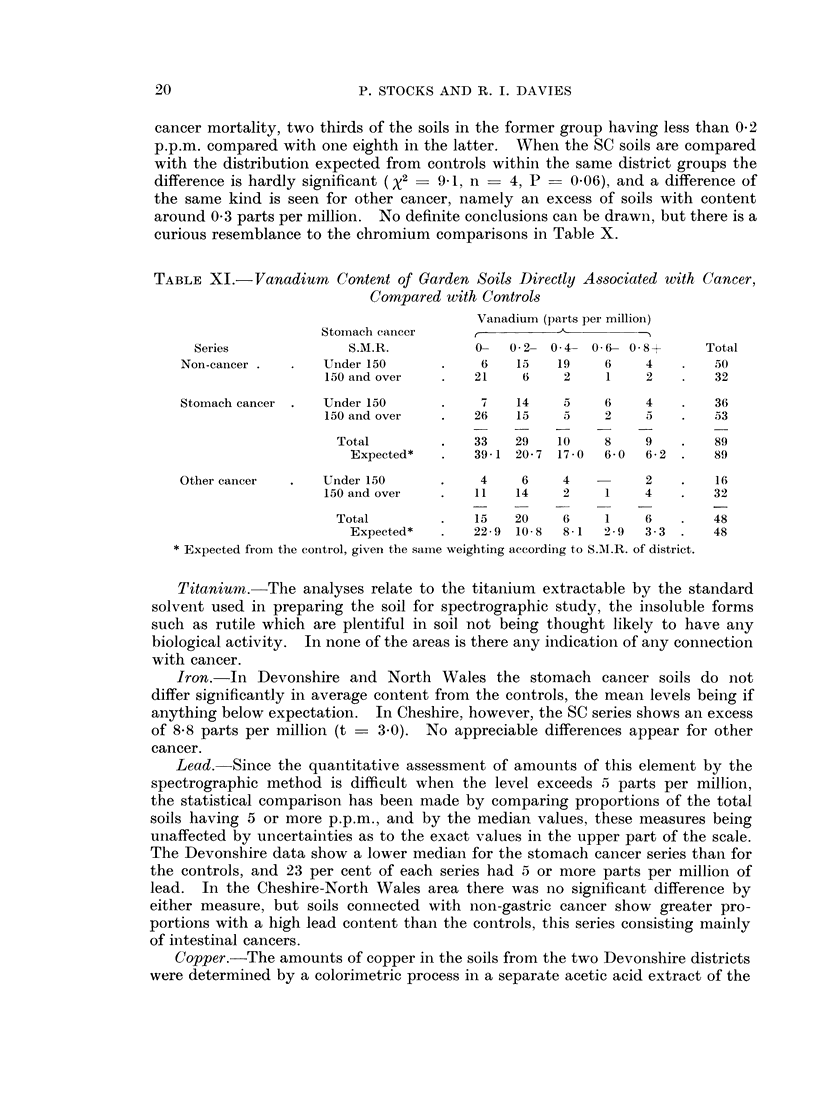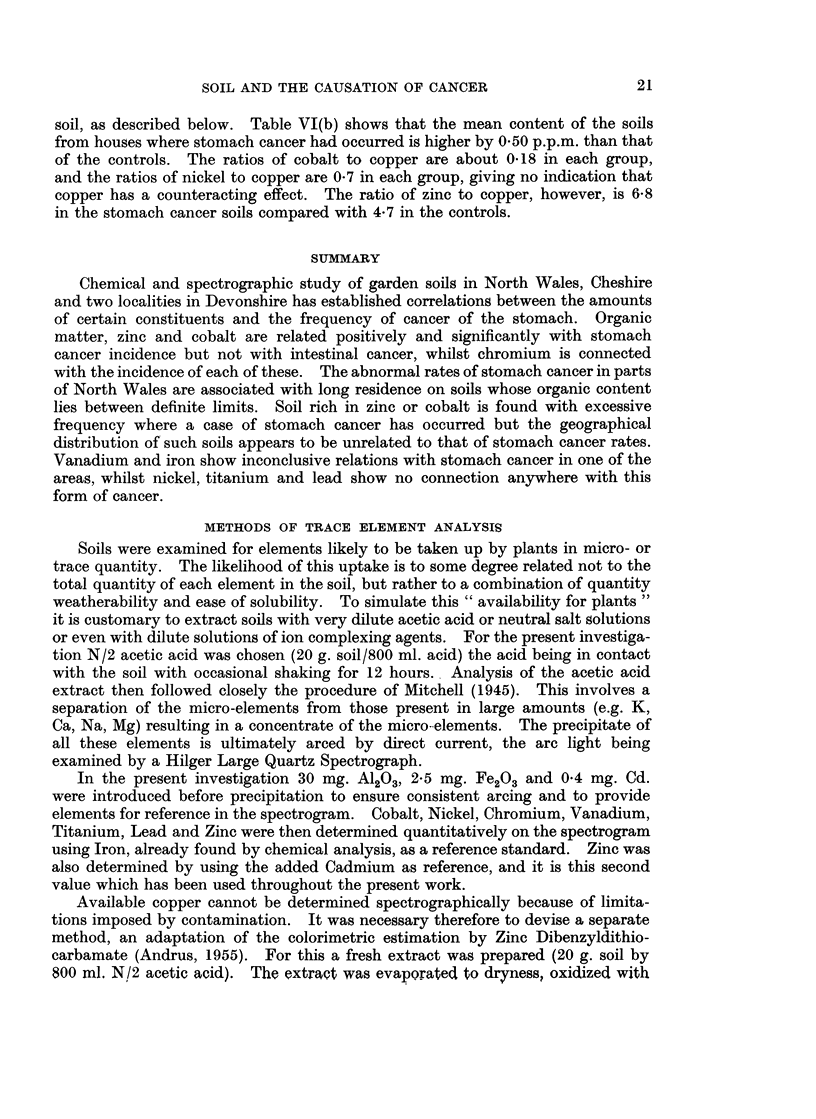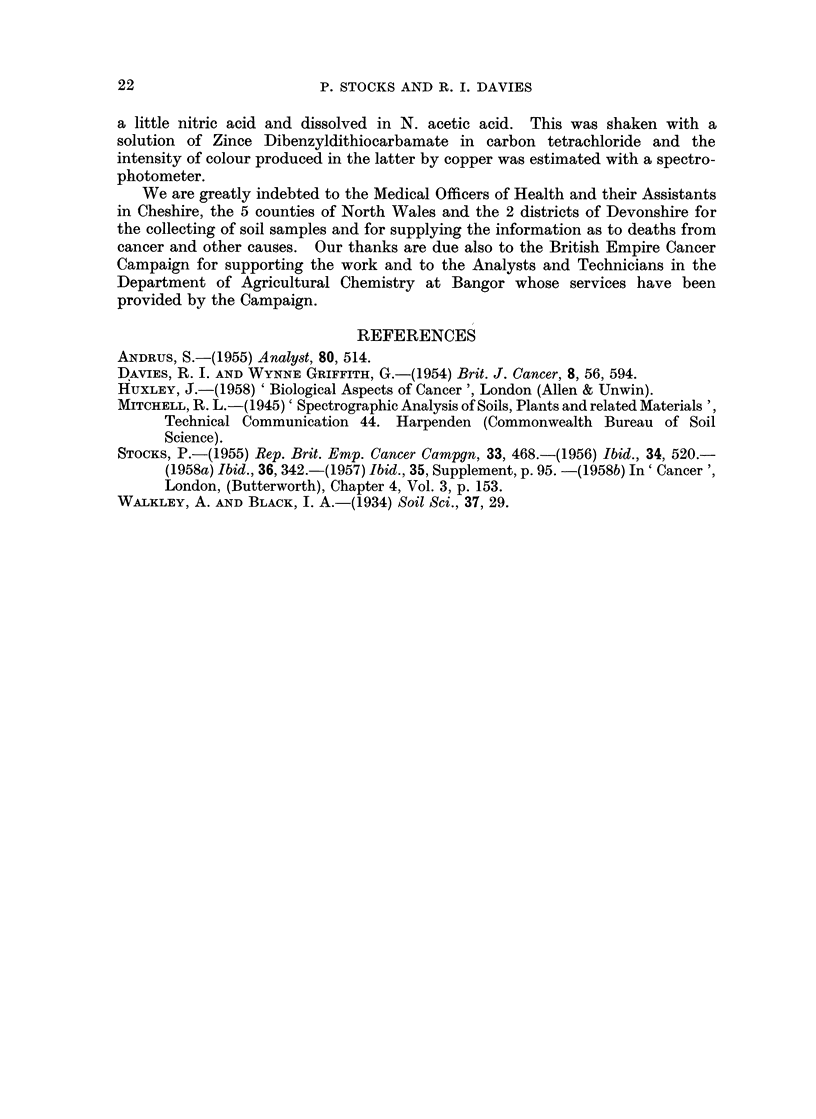# Epidemiological Evidence from Chemical and Spectrographic Analyses that Soil is Concerned in the Causation of Cancer

**DOI:** 10.1038/bjc.1960.2

**Published:** 1960-03

**Authors:** P. Stocks, R. I. Davies


### EPIDEMIOLOGICAL EVIDENCE FROM CHEMICAL AND SPECTROGRAPHIC ANALYSES THAT SOIL IS CONCERNED IN THE CAUSATION OF CANCER

P. STOCKS AND R. I. DAVIES

*From the Department of Agricultural Chemistry, University College of North Wales, Bangor*

Received for publication December 5, 1959

THE reasons which led to the initiation in 1952 of a study of the chemical properties of garden soils in connection with cancer in North Wales and Cheshire have been detailed (Stocks, 1957, 1958). The Cheshire and North Wales Branch of the Campaign had included soil amongst the environmental items to be investigated in the 4-year cancer survey, and with their help the Department of Agricultural Chemistry was enabled to undertake the chemical analyses, and at a later date spectrographic estimations of trace elements. Collection of soil samples from gardens of houses where a death of a resident had occurred from cancer or other cause was made possible by the co-operation of the Medical Officers of Health and was carried out by their Public Health Inspectors.

Since 1957 the laboratory work on some 2,000 soils has continued with the aid of a grant to the University College of North Wales, a special study has been made also of two districts with high and low incidence of stomach cancer in Devonshire, and analytical work on plants and spectroscopic study of polycyclic hydrocarbons have been started. Brief progress reports have appeared in the Annual Reports of the British Empire Cancer Campaign for 1955 to 1958, and the purpose of this paper is to present a more complete and up-to-date account of the outcome so far of the work on soils, including the Devonshire study.

A hypothesis that one of the causes of cancer of the stomach is in some way connected with soil cannot be dismissed on the grounds that we do not know how such a connection could arise. Kant once remarked that " Philosophy is often much embarrassed when she encounters certain facts which she dare not doubt yet will not believe for fear of ridicule ". The soil hypothesis has met with scepticism from those who look for a single " cause " of cancer, but as Sir Julian Huxley (1958) wrote in discussing the statistical evidence about lung cancer, " It is surely time that we should drop mediaeval concepts concerning causation and think in terms of multiple correlation ". Having established as a first step the existence of statistical correlations between the incidence of stomach cancer and the amounts of organic material and certain trace elements in the neighbouring soil. possible reasons for this such as vegetables, water supply, air pollution and radioactivity have next to be investigated.

### *Organic Content of Soil in Districts having Different Rates of Stomach Cancer Mortality*

In the North Wales and Chesire region where soils contain inconsiderable amounts of free calcium carbonate the loss of weight on ignition of a dry soil, expressed as a percentage, provided an index of the organic matter present. Where the soils are contaminated with coal or ashes the loss on ignition would be affected, so a second index was obtained of the readily oxidisable or "organic carbon" by a wet chemical oxidation of the soil (Walkley and Black, 1934). In this method, coal and ash are but little affected and so do not contribute to the index. Soils seen on examination in the dried state to be appreciably contaminated by small coal have, however, been dealt with separately in the statistical analysis.

During the years whilst soil samples were being collected the aim was to obtain a sample from every house where a death occurred from cancer of the stomach, intestine, rectum, larynx, lung or breast or from leukaemia or Hodgkin's disease, and in the counties of Anglesey, Caernarvon and Cheshire omissions arose only through absence of a garden or inability to contact relatives or obtain the necessary permission. In the other counties the coverage was not so complete owing to pressure of work on the Health Officers, but such selection of cases as resulted was connected with periods of time and not with the nature of the cases. Histories of all persons dying of cancer were obtained independently from relatives if no hospital history had been obtained previously, and this information included the duration of residence at the house prior to death. In order to obtain a control series addresses were taken from the monthly lists of deaths from causes other than cancer by matching the sex, age and district of residence with the first 600 stomach cancer cases as nearly as was practicable.

The soil study covered the 55 administrative areas in North Wales and 11 in the parts of Cheshire within the Liverpool Hospital Region, excluding the county boroughs, and details of these were given by Stocks (1957, Table 6 and Map 2).

The standardised mortality ratios (S.M.R.'s) for cancer of the stomach in the 8-year period 1947–55 were given in the final column of that table and depicted in Map 3, and they ranged from 65 to 262 compared with 100 for England and Wales, four very small districts with fewer than 5 deaths being combined with adjoining areas. On page 104 of the report a general similarity between the geographical distribution of stomach cancer mortality and organic carbon content of soils not directly associated with cases of cancer was pointed out, whereas no such correspondence was apparent with cancer mortality rates for intestine, lung or breast.

When the districts are divided up into 9 groups with stomach cancer S.M.R. 50–, 75–, 100–, 125–, 150–, 175–, 200–, 225–, 250–274, and soils distributed on scales of organic carbon and ignition loss, the correlation coefficients between organic content and the mortality from stomach cancer in the district where the soil was taken are as shown in Table I.

TABLE I.—*Correlations Between Measures of Organic Matter Content of Soils not Directly Connected with a Case of Stomach Cancer and the Level of Mortality from Stomach Cancer in the District from which the Soil was taken, Estimated from Two Alternative Series of Samples*

| | (1) Non-cancer control soils | | | (2) Non-gastric cancer soils | | | Correlation with S.M.R. from stomach cancer in | |
|---|---|---|---|---|---|---|---|---|
| | Number | Mean | S.D. | Number | Mean | S.D. | 1st Series | 2nd series |
| Organic carbon | 513 | 2·98 | 1·22 | 1063 | 2·86 | 1·35 | 0·4661 | 0·4015 |
| Ignition loss* | 373 | 9·34 | 4·12 | 681 | 9·24 | 4·09 | 0·5693 | 0·5027 |

* When the ash-contaminated soils are included the coefficients become 0·481 in the non-cancer series and 0·462 in the other series.

TABLE II.—*Organic Carbon Content of Soils from Houses with Stomach Cancer, showing Excess over the Calculated Frequencies based on the Corresponding Distributions of Other Cancer and Non-cancer Cases (corrected for the Relative Mortality from Stomach Cancer in the District).*

| Years in house | Organic carbon per 100 | No. of soils grouped according to S.M.R. of district: 50– | 100– | 125– | 150– | 175– | 200+ | All | Sub-totals: 50– | 150+ | Excess as calculated from: Non- 50– | Other 50– | Non- 150+ | Other 150+ |
|---|---|---|---|---|---|---|---|---|---|---|---|---|---|---|
| Under 10 | 0– | 18 | 18 | 7 | 3 | 3 | 2 | 51 | 43 | 8 | 7·1 | 6·5 | 1·8 | −1·4 |
| | 2– | 12 | 19 | 15 | 18 | 5 | 12 | 81 | 46 | 35 | −12·1 | −5·6 | 15·6 | 21·4 |
| | 3·5– | 4 | 4 | 7 | 8 | 6 | 4 | 33 | 15 | 18 | 4·8 | −1·0 | 8·5 | 7·3 |
| | 5+ | — | 1 | 4 | 5 | 2 | 4 | 16 | 5 | 11 | 3·0 | 3·4 | 6·8 | 5·6 |
| | Total | 34 | 42 | 33 | 34 | 16 | 22 | 181 | 109 | 72 | 2·8 | 3·3 | 32·7 | 32·9 |
| 10–19 | 0– | 11 | 8 | 7 | 5 | 2 | 2 | 35 | 26 | 9 | 1·1 | −6·6 | 6·1 | 3·1 |
| | 2– | 19 | 19 | 8 | 16 | 3 | 6 | 71 | 46 | 25 | 4·5 | 3·0 | 3·5 | 10·6 |
| | 3·5– | 2 | 3 | 4 | 8 | 6 | 6 | 29 | 9 | 20 | −9·0 | −0·4 | 9·2 | 5·5 |
| | 5+ | — | 5 | 4 | 5 | 4 | 9 | 27 | 9 | 18 | 4·0 | 4·7 | 15·2 | 14·1 |
| | Total | 32 | 35 | 23 | 34 | 15 | 23 | 162 | 90 | 72 | 0·6 | 0·7 | 34·0 | 33·3 |
| 20 and over | 0– | 13 | 8 | 8 | 2 | — | — | 31 | 29 | 2 | 13·0 | −8·8 | −4·2 | −6·6 |
| | 2– | 16 | 21 | 19 | 37 | 16 | 17 | 126 | 56 | 70 | −18·6 | 4·4 | 42·5 | 44·4 |
| | 3·5– | 5 | 11 | 14 | 26 | 13 | 13 | 82 | 30 | 52 | 0·7 | 2·0 | 9·0 | 18·6 |
| | 5+ | 4 | 4 | 9 | 10 | 4 | 11 | 42 | 17 | 25 | 13·0 | 8·0 | 17·1 | 10·2 |
| | Total | 38 | 44 | 50 | 75 | 33 | 41 | 281 | 132 | 149 | 8·1 | 5·6 | 64·4 | 66·6 |

Organic carbon distributions of soils from houses in same groups of districts where a death had ocurred from :

| Years in house | Organic carbon per 100 | Non-cancer cause: 50– | 100– | 125– | 150– | 175– | 200+ | All | Other cancer (i.e. not stomach): 50– | 100– | 125– | 150– | 175– | 200+ | All |
|---|---|---|---|---|---|---|---|---|---|---|---|---|---|---|---|
| Under 10 | 0– | 6 | 6 | 9 | 4 | 2 | 2 | 29 | 39 | 33 | 10 | 12 | 6 | 2 | 102 |
| | 2– | 11 | 9 | 9 | 14 | 10 | 4 | 57 | 36 | 45 | 33 | 16 | 8 | 18 | 156 |
| | 3·5– | 1 | 2 | 2 | 6 | 5 | 6 | 22 | 7 | 13 | 18 | 15 | 7 | 13 | 73 |
| | 5+ | 1 | — | — | 3 | 2 | 1 | 7 | 1 | — | 3 | 5 | 2 | 10 | 21 |
| | Total | 19 | 17 | 20 | 27 | 19 | 13 | 115 | 83 | 91 | 64 | 48 | 23 | 43 | 352 |
| 10–19 | 0– | 10 | 1 | 2 | 3 | — | — | 16 | 40 | 17 | 8 | 5 | 3 | 2 | 75 |
| | 2– | 12 | 2 | 10 | 10 | 10 | 6 | 50 | 33 | 32 | 22 | 16 | 9 | 9 | 121 |
| | 3·5– | 4 | 1 | 2 | 8 | 3 | 2 | 21 | 4 | 6 | 11 | 12 | 7 | 3 | 43 |
| | 5+ | — | 1 | — | 2 | — | 1 | 4 | 3 | 1 | 6 | 3 | 1 | 4 | 18 |
| | Total | 26 | 6 | 14 | 23 | 13 | 9 | 91 | 80 | 56 | 47 | 36 | 20 | 18 | 257 |
| 20 and over | 0– | 9 | 2 | 2 | 1 | 2 | 3 | 19 | 32 | 21 | 13 | 4 | 7 | 3 | 80 |
| | 2– | 18 | 11 | 14 | 20 | 8 | 4 | 75 | 34 | 32 | 28 | 27 | 7 | 10 | 138 |
| | 3·5– | 4 | 6 | 11 | 33 | 10 | 10 | 74 | 10 | 11 | 24 | 37 | 15 | 5 | 102 |
| | 5+ | — | — | 3 | 5 | 3 | 2 | 13 | 3 | 5 | 8 | 11 | 2 | 11 | 40 |
| | Total | 31 | 19 | 30 | 59 | 23 | 19 | 181 | 79 | 69 | 73 | 79 | 31 | 29 | 360 |

TABLE III.—*Loss on Ignition of Dried Soils from Houses with Stomach Cancer, Showing Excess over the Calculated Frequencies based on the corresponding Distributions of Other Cancer and Non-cancer Cases (Corrected for the Relative Mortality from Stomach Cancer in the District)*

| Years in house | Loss on ignition per 100 | No. of soils grouped according S.M.R. of district: 50– | 100– | 125– | 150– | 175– | 200+ | All | Sub-totals: 50– | 150+ | Excess as calculated from: Non- 50– | Other 50– | Non- 150+ | Other 150+ |
|---|---|---|---|---|---|---|---|---|---|---|---|---|---|---|
| Under 10 | 0– | 24 | 25 | 11 | 8 | 3 | 3 | 74 | 60 | 14 | −6·7 | 0·6 | 0·7 | 5·2 |
| | 8·5– | 7 | 12 | 7 | 9 | 3 | 3 | 41 | 26 | 15 | 0·1 | −0·6 | 6·6 | 2·8 |
| | 11·5– | 4 | 2 | 8 | 7 | 4 | 3 | 28 | 14 | 14 | 3·0 | 0·1 | 5·4 | 6·4 |
| | 14·5+ | 2 | 3 | 10 | 10 | 6 | 13 | 44 | 15 | 29 | 6·4 | 3·1 | 19·9 | 16·6 |
| | Total | 37 | 42 | 36 | 34 | 16 | 22 | 187 | 115 | 72 | 2·8 | 3·2 | 32·6 | 31·3 |
| 10–19 | 0– | 23 | 20 | 9 | 11 | 2 | 6 | 71 | 52 | 19 | −2·1 | 2·4 | 13·3 | 12·3 |
| | 8·5– | 6 | 9 | 3 | 9 | 1 | 5 | 33 | 18 | 15 | −2·1 | −7·2 | −6·7 | 4·9 |
| | 11·5– | 4 | 5 | 3 | 5 | 5 | 4 | 26 | 12 | 14 | 3·8 | 3·8 | 2·0 | 3·3 |
| | 14·5+ | 1 | 4 | 8 | 10 | 8 | 8 | 39 | 13 | 26 | 0·7 | 1·5 | 11·6 | 13·2 |
| | Total | 34 | 38 | 23 | 35 | 16 | 23 | 169 | 95 | 74 | 0·3 | 0·5 | 33·6 | 33·7 |
| 20 and over | 0– | 19 | 8 | 11 | 9 | 2 | 3 | 52 | 38 | 14 | −14·2 | −16·9 | 6·7 | 5·7 |
| | 8·5– | 7 | 15 | 10 | 17 | 6 | 7 | 62 | 32 | 30 | −0·4 | 6·9 | 11·9 | 13·9 |
| | 11·5– | 7 | 11 | 11 | 22 | 12 | 19 | 82 | 29 | 53 | 8·9 | 24·1 | 31·6 | 37·9 |
| | 14·5+ | 5 | 6 | 16 | 29 | 13 | 12 | 81 | 27 | 54 | 7·8 | 1·5 | 17·3 | 10·1 |
| | Total | 38 | 40 | 48 | 77 | 33 | 41 | 277 | 126 | 151 | 2·1 | 5·6 | 67·5 | 67·5 |

Ignition loss distributions of soils from houses in same groups of districts where a death had occurred from :

| Years in house | Loss on ignition per 100 | Non-cancer cause: 50– | 100– | 125– | 150– | 175– | 200+ | All | Other cancer (i.e. not stomach): 50– | 100– | 125– | 150– | 175– | 200+ | All |
|---|---|---|---|---|---|---|---|---|---|---|---|---|---|---|---|
| Under 10 | 0– | 19 | 15 | 11 | 10 | 3 | 5 | 63 | 59 | 41 | 17 | 9 | 5 | 6 | 137 |
| | 8·5– | 10 | 2 | 5 | 7 | 3 | 2 | 29 | 10 | 25 | 21 | 12 | 9 | 6 | 83 |
| | 11·5– | 2 | 2 | 3 | 6 | 6 | 2 | 21 | 5 | 14 | 11 | 7 | 4 | 11 | 52 |
| | 14·5+ | 2 | 2 | 1 | 5 | 7 | 2 | 19 | 6 | 4 | 14 | 20 | 5 | 21 | 70 |
| | Total | 33 | 21 | 20 | 28 | 19 | 11 | 132 | 80 | 84 | 63 | 48 | 23 | 44 | 342 |
| 10–19 | 0– | 22 | 9 | 6 | 5 | 1 | — | 43 | 53 | 23 | 8 | 4 | 4 | 3 | 95 |
| | 8·5– | 5 | 3 | 4 | 4 | 3 | 2 | 21 | 14 | 18 | 14 | 10 | 7 | 3 | 66 |
| | 11·5– | 4 | — | 3 | 4 | 6 | 4 | 21 | 2 | 5 | 13 | 10 | 4 | 5 | 39 |
| | 14·5+ | 1 | 2 | 3 | 8 | 3 | 3 | 20 | 4 | 6 | 15 | 13 | 7 | 8 | 53 |
| | Total | 32 | 14 | 16 | 21 | 13 | 9 | 105 | 73 | 52 | 50 | 37 | 22 | 19 | 201 |
| 20 and over | 0– | 34 | 10 | 6 | 4 | 3 | 3 | 60 | 55 | 34 | 16 | 4 | 5 | 5 | 119 |
| | 8·5– | 8 | 10 | 10 | 15 | 4 | 3 | 50 | 14 | 19 | 12 | 15 | 5 | 7 | 72 |
| | 11·5– | 5 | 4 | 5 | 15 | 4 | 5 | 38 | 5 | 8 | 12 | 14 | 6 | 5 | 50 |
| | 14·5+ | 6 | 2 | 8 | 25 | 11 | 8 | 60 | 8 | 6 | 29 | 47 | 13 | 11 | 114 |
| | Total | 53 | 26 | 29 | 59 | 22 | 19 | 208 | 82 | 67 | 69 | 80 | 29 | 28 | 355 |

There is an unmistakable relation between the average amount of organic material in the soil samples taken throughout a district and the mortality there from stomach cancer, but the regression of the latter upon the former is not a simple one. As shown below, the association between the factors is very pronounced in the part of the scale where the index lies between 2 and 3¼ :—

| | | | | | | | | | |
|---|---|---|---|---|---|---|---|---|---|
| Organic carbon index . . . | 1¼ | | 2¼ | | 3¼ | | 4¼ | | 5¼ |
| Mean stomach cancer S.M.R. . . | 116 | | 123 | | 160 | | 170 | | 181 |
| Increment of S.M.R. per unit . . | | 7 | | 37 | | 10 | | 11 | |

This accords with the finding in the next section that stomach cancer cases occurred with peculiar frequency after long residence in houses where the soil contained between 2·0 and 3·5 parts per 100 of organic carbon. Soils of the " $\beta$ " group described by Davies and Wynne Griffith (1954) and found by them to be associated significantly with stomach cancer deaths in Anglesey also tend to have an organic carbon content about this magnitude.

*Organic Content of Garden Soils Directly Associated with Cancer*

Soil samples were obtained from gardens of houses where a death from stomach cancer had occurred, and 690 of these have been analysed for organic carbon and 700 for loss on ignition. Information as to length of residence at the house before onset of the illness was available for 92 per cent of these and Tables II and III show the distributions on scales of organic carbon and ignition loss for durations of under 10, 10–19 and 20 or more years, with subdivision of the districts into 6 groups according to their stomach cancer mortality in 1947–54. The lower parts of the tables show the corresponding distributions of alternative control series of soils taken at houses where a death had occurred (1) from a non-malignant cause and (2) from non-gastric cancer. In calculating the expected frequencies for comparison with the stomach cancer numbers the soils were further sub-divided into those with and without ash contamination, each being calculated separately. The aggregated result for each district group was then divided by the stomach cancer mortality ratio to obtain the expected frequency distribution if stomach cancer incidence were uniform and unrelated to soil characters. The divergencies from expectation in the right hand columns of the upper parts of the tables show, therefore, how the stomach cancer cases *in excess of the normal* were distributed on the soil scales, the " normal " being derived in two ways, firstly from a matched control series where cancer was not known to have occurred and secondly from the unselected series associated with cancers of the intestine, lung, breast and a few other sites.

In districts where the incidence of stomach cancer is below or or not greatly above the national average (S.M.R. 50–149) a condensed comparison between the actual and expected distributions of soils from houses where stomach cancer occurred is shown in Table IV.

The only differences noticeable here are a concentration of the stomach cases on soils with organic carbon exceeding 5 (31 compared with a mean expectation of 12), and a tendency for the stomach cases to occur where the soil has an ignition loss of 11·5 or more (110 compared with a mean expectation of 82·5). It is evident that in the districts with not very abnormal stomach cancer incidence there is a tendency for high content of organic material in the garden soil to be associated with occurrence of stomach cancer in the house.

TABLE IV.—*Actual and Expected Organic Content of Garden Soils Associated with Stomach Cancer in Districts with S.M.R. Below* 150

| | Organic carbon | | | | Ignition loss (per cent) | | | | |
|---|---|---|---|---|---|---|---|---|---|
| | 0– | 3·5– | 5+ | Total | 0– | 8·5– | 11·5– | 14·5+ | Total |
| | Resident less than 20 years in house | | | | | | | | |
| Actual cases | 161 | 24 | 14 | 199 | 112 | 44 | 26 | 28 | 210 |
| Expected from Non-cancer | 160·4 | 28·2 | 7·0 | 195·6 | 120·8 | 46·0 | 19·2 | 20·9 | 206·9 |
| Expected from Other cancer | 163·7 | 25·4 | 5·9 | 195·0 | 109·0 | 51·8 | 22·1 | 23·4 | 206·3 |
| | Resident 20 years or more in house | | | | | | | | |
| Actual cases | 85 | 30 | 17 | 132 | 38 | 32 | 29 | 27 | 126 |
| Expected from Non-cancer | 90·6 | 29·3 | 4·0 | 123·9 | 52·2 | 32·4 | 20·1 | 19·2 | 123·9 |
| Expected from Other cancer | 89·4 | 28·0 | 9·0 | 126·4 | 54·9 | 25·1 | 14·9 | 25·5 | 120·4 |

In the aggregate of districts where mortality from stomach cancer is very high (S.M.R. 150–262), if the large excess of cases over the normal expectation is unconnected with soil characters its distribution on the soil scales should not differ significantly from the distribution of the expected cases, and if there is a concentration of the excess at a particular point in the soil scale that would support the hypothesis that a soil factor accounts for the abnormally high incidence of stomach cancer in parts of North Wales, this being superimposed on the other causes which operate throughout England and Wales. The comparison summarised in Table V shows amongst people who had lived less than 20 years in the house a strong concentration of the stomach cancer cases on soils with high organic carbon and ignition loss, similar to that found in the more normal districts (Table IV). For people who had lived 20 years or more in the house and then died of cancer or other cause, the surplus of stomach cancer is distributed

TABLE V.—*Actual and Expected Organic Content of Garden Soils Associated with Stomach Cancer in Districts with High S.M.R.* (150+)

| | Organic carbon | | | | | Ignition loss (per cent) | | | | |
|---|---|---|---|---|---|---|---|---|---|---|
| | 0– | 2– | 3·5– | 5+ | Total | 0– | 8·5– | 11·5– | 14·5+ | Total |
| | Resident less than 20 years in house | | | | | | | | | |
| Actual cases | 17 | 60 | 38 | 29 | 144 | 33 | 30 | 28 | 55 | 146 |
| Expected from Non-cancer | 9·1 | 40·9 | 20·3 | 7·0 | 77·3 | 19·2 | 16·7 | 20·6 | 23·3 | 79·8 |
| Expected from Other cancer | 15·3 | 28·0 | 25·2 | 9·3 | 77·8 | 15·2 | 22·3 | 18·3 | 25·2 | 81·0 |
| Excess (per cent) over mean expectation | 39 | 74 | 67 | 256 | 86 | 92 | 54 | 44 | 127 | 82 |
| Per cent of total | | | | | | | | | | |
| Mean expected number | 15·7 | 44·4 | 29·4 | 10·5 | 100·0 | 21·4 | 24·2 | 24·2 | 30·2 | 100·0 |
| Excess cases | 7·2 | 38·4 | 23·0 | 31·4 | 100·0 | 24·1 | 16·0 | 13·0 | 46·9 | 100·0 |
| | Resident 20 years or more in house | | | | | | | | | |
| Actual cases | 2 | 70 | 52 | 25 | 149 | 14 | 30 | 53 | 54 | 151 |
| Expected Non-cancer | 6·2 | 27·5 | 43·0 | 7·9 | 84·6 | 7·3 | 18·1 | 21·4 | 36·7 | 83·5 |
| Expected Other cancer | 7·4 | 26·6 | 38·2 | 11·3 | 83·5 | 8·3 | 16·1 | 15·2 | 43·9 | 83·5 |
| Excess (per cent) over mean expectation | −73 | 164 | 36 | 120 | 78 | 79 | 70 | 190 | 34 | 81 |
| Per cent of total | | | | | | | | | | |
| Mean expected number | 8·1 | 32·2 | 48·3 | 11·4 | 100·0 | 9·3 | 20·5 | 21·9 | 48·3 | 100·0 |
| Excess cases | −7·4 | 66·1 | 17·6 | 23·7 | 100·0 | 9·2 | 19·1 | 51·4 | 20·3 | 100·0 |

on the soil scales in a manner very different from that expected. Two thirds of the excess occurs on soils with organic carbon between 2 and 3·5 per 100 compared with one third of the control, and one half occur on soils with ignition loss between 11·5 and 14·5 per cent compared with about one fifth of the control. These differences from expectation are highly significant from a probability standpoint, and they seem to indicate that long residence on soil of this special type in North Wales is peculiarly conducive to cancer of the stomach, the connection with soils of high organic content being more pronounced where there was appearance of stomach cancer after shorter periods of residence.

In Table VI(*b*) the mean organic carbon and ignition loss of soils from 43 houses where a death from stomach cancer (SC) had occurred in two Devonshire townships are compared with a control series taken from corresponding subdistricts of those areas The SC series gives higher mean values than the control. (N) both for organic carbon and for ignition loss. Furthermore, as shown in Table VI(*a*), the general level of organic matter in the soil is higher in the locality with a high death rate from stomach cancer than in the locality with a low rate. These results, obtained by a more rigid control method than was possible in North Wales, agree with what has been found there.

*Trace Elements in Garden Soils in North Wales, Cheshire and Devonshire Districts with High and Low incidence of Stomach Cancer*

Two neighbouring and similar Devonshire townships A and B each with about 4,000 people, have shown over the last 10 years very different death rates from cancer of the stomach, the S.M.R's being estimated at 187 and 52 respectively. Owing to dissemination at A of industrial waste containing mineral substances which could be carcinogenic, a comparison of garden soils in A and B was carried out with the co-operation of the County Health Officers in respect of the trace element contents, using the spectrographic method. Each place was divided into 24 sub-areas and in each of these three gardens were chosen at random, except that addresses where cancer was known to have occurred were avoided, soil samples being taken and mixed together producing 24 samples representative of the sub-areas in A, and likewise in B. The dried soils were analysed spectrographically for 7 elements and chemically for iron and copper by the methods described on page 20. The mean values in parts per million for the two places are shown in Table VI(*a*).

The average amounts of 7 of the elements were greater in the soils from A where the stomach cancer mortality is high than in those from B where it is low, the excess being highly significant for cobalt, nickel and iron. Neither in district A nor in B are the values necessarily representative of Devonshire soils in general, and no useful comparison with the averages for North Wales and Cheshire in Table VII can be made.

In the process of obtaining a control series of garden soils for trace element analysis 36 from parts of North Wales where the incidence of stomach cancer is abnormally high and 48 from Cheshire where it is normal were chosen from the addresses where a person had died of a cause other than cancer after 10 years or more of residence in the house. Comparison of the results of spectrographic analysis in respect of 8 elements is shown in Table VII, the method used being the same as for the Devon soils (p. 20).

TABLE VI.—*Trace Elements and Organic Matter in Garden Soils of Two Devonshire Townships*

(*a*) Comparison between soils randomly taken from A where mortality from stomach cancer is high and from B where the mortality is low.

| | Number in each place | Mean content in soils from A | Mean content in soils from B | Difference A − B | Per cent of combined average |
|---|---|---|---|---|---|
| Organic carbon (per cent) | 72 | 3·22 | 2·82 | 0·40 | 13 |
| Ignition loss (per cent) | 72 | 11·07 | 7·90 | 3·17 | 29 |
| Copper (parts per million) | 72 | 2·62 | 1·95 | 0·67 | 25 |
| Zinc ,, ,, ,, | 72 | 115 | 120 | −5 | .. |
| Chromium ,, ,, ,, | 72 | 0·215 | 0·192 | 0·023 | 11 |
| Cobalt ,, ,, ,, | 72 | 0·462 | 0·326 | 0·136 | 35 |
| Nickel ,, ,, ,, | 72 | 1·68 | 1·17 | 0·51 | 36 |
| Vanadium ,, ,, ,, | 72 | 0·286 | 0·362 | −0·076 | .. |
| Titanium ,, ,, ,, | 72 | 0·190 | 0·153 | 0·037 | 22 |
| Iron ,, ,, ,, | 72 | 24·9 | 17·7 | 7·2 | 34 |
| Lead (median values in p.p.m.) | 72 | 3·7 | 2·9 | 0·8 | 24 |

(*b*) Comparison between soils from houses where stomach cancer had occurred (SC) and a control series from the same sub-districts of A and B (N).

| | Number in each group | Mean content in soils of series SC | Mean content in soils of series N | Difference SC − N | Ratio to standard error |
|---|---|---|---|---|---|
| Organic carbon (per cent) | 43 | 3·46 | 3·02 | 0·44 | 1·4 |
| Ignition loss (per cent) | 43 | 11·90 | 9·96 | 1·94 | 1·7 |
| Copper (parts per million) | 44 | 2·68 | 2·18 | 0·50 | 1·5 |
| Zinc ,, ,, ,, | 44 | 181 | 103 | 78 | 2·8 |
| Chromium ,, ,, ,, | 44 | 0·289 | 0·193 | 0·096 | 2·8 |
| Cobalt ,, ,, ,, | 44 | 0·502 | 0·371 | 0·131 | 2·5 |
| Nickel ,, ,, ,, | 44 | 1·93 | 1·48 | 0·45 | 2·2 |
| Vanadium ,, ,, ,, | 44 | 0·407 | 0·303 | 0·104 | 2·0 |
| Titanium ,, ,, ,, | 44 | 0·132 | 0·170 | −0·038 | .. |
| Iron ,, ,, ,, | 44 | 19·5 | 21·8 | −2·3 | .. |
| Lead (median values in p.p.m.) | 44 | 2·05 | 3·50 | −1·45 | .. |

TABLE VII.—*Trace Element Content of Garden Soils not Directly Connected with Cancer in North Wales and Cheshire*

| | Mean content (p.p.m.) in North Wales (36) | Mean content (p.p.m.) in Cheshire (48) | Difference | Ratio to its standard error |
|---|---|---|---|---|
| Zinc | 51·2 | 54·3 | −3·1 | 0·2 |
| Chromium | 0·230 | 0·178 | +0·052 | 2·0 |
| Cobalt | 0·522 | 0·528 | −0·006 | 0·1 |
| Nickel | 1·046 | 1·230 | −0·184 | 1·8 |
| Titanium (soluble) | 0·228 | 0·213 | +0·015 | 0·7 |
| Vanadium | 0·342 | 0·455 | −0·113 | 2·1 |
| Iron | 23·8 | 20·7 | +3·1 | 1·2 |
| Lead (median values) | 2·1 | 5·2 | −3·1 | .. |

The North Wales soils show an excess of chromium, whilst nickel, vanadium and lead are more plentiful in the Cheshire soils. There is no indication from these comparisons that the general level of zinc is any greater in districts with high incidence of cancer of the stomach than it is elsewhere, and for cobalt this was the case in the Devonshire townships investigated but not in the Cheshire and North Wales area. This has to be kept in mind when seeking an explantion of the results recorded in the next section. The excess of chromium, cobalt and nickel in locality A (Table VI*a*) may be of industrial origin and investigation of this possibility is not yet complete.

*Trace Elements in Garden Soils Directly Associated with Cancer*

In the Devonshire township A, already referred to, 37 deaths from cancer of the stomach had occurred in 15 years, and soil samples were taken from the gardens of houses where the deceased persons had last lived, the number of years residence there before death being ascertained when possible. Within township B 7 such deaths had occurred and samples were taken similarly. Each of the 44 cases was matched by a non-cancer sample from the same sub-area, and Table VI(*b*) compares the mean trace element contents of the soils in the stomach cancer (SC) and non-cancer (N) series.

In North Wales spectrographic examination has been made of 73 soils from houses in the 5 counties where a resident had died of stomach cancer after living there 10 years or more, and of 39 soils similarly associated with a death from cancer of the intestine, lung or breast, the frequencies of these being 29, 7 and 3 respectively. From the Cheshire areas, comprising the Wirral, Runcorn, Lymm and the rural districts of Chester and Tarvin, 31 soils associated with stomach cancer and 9 associated with intestinal cancer have been analysed. The mean trace element contents of these groups (SC and OC) are compared in Table VIII with those for the non-cancer control series (N).

*Zinc.*—In North Wales the stomach cancer soils show a wide range of values from 4 to 441 part per million, with a mean of 81·2 compared with 51·2 for the non-cancer controls, the excess of 30·0 being twice its standard error. In Cheshire the SC soils show a range from 3 to 387 with a mean of 83·4 compared with 54·3 for the controls, the excess of 29·1 being $1\frac{1}{2}$ times its standard error. In the combined area the excess of 28·8 is 2·4 times its standard error. In the two Devon localities where the zinc levels happen to be higher the mean for the SC soils is 181 compared with 103 for the matched controls, giving an excess 2·8 times its standard error.

The odds against finding such agreement in three independent series by chance, with $t = 2{\cdot}0$, 1·5 and 2·8, are enormously great, and it must be concluded that a zinc content of the soil higher than the local average is a factor favourable to the appearance of stomach cancer, and that this is not confined to districts where the general incidence is specially high. Indeed, as shown in Tables VI and VII, there is no tendency for districts with high stomach cancer mortality to have higher zinc levels in the soils as a whole than districts with low mortality, and yet in the gardens of houses where deaths from stomach cancer occurred the zinc level is higher than in other gardens of the same area. This seems to indicate that another factor must be present which acts in conjunction with zinc and which is more plentiful in soils of districts where stomach cancer incidence is high than

TABLE VIII.—*Quantities of Trace Elements (parts per million) in Soils from Gardens of Houses in North Wales and Cheshire where a Death had Occurred from Cancer of the Stomach (SC), Other Cancer (OC) or from a Non-cancer cause (N)*

| | | North Wales* | | | Cheshire* | | | Combined area* | | |
|---|---|---|---|---|---|---|---|---|---|---|
| | | No. | Mean (p.p.m.) | Diff. (C − N) | No. | Mean (p.p.m.) | Diff. (C − N) | Mean (p.p.m.) | Diff. (C − N) | S.E. |
| Zinc | N | 36 | 51·2 | .. | 48 | 54·3 | .. | 53·0 | .. | .. |
| | SC | 73 | 81·2 | +30·0 | 31 | 83·4 | +29·1 | 81·8 | +28·8 | 12·0 |
| | OC | 39 | 64·9 | +13·7 | 9 | 56·1 | +1·8 | 63·2 | +10·2 | 11·8 |
| Cobalt | N | 36 | 0·522 | .. | 48 | 0·528 | .. | 0·525 | .. | .. |
| | SC | 73 | 0·655 | +0·133 | 31 | 0·625 | +0·097 | 0·646 | +0·121 | 0·049 |
| | OC | 39 | 0·583 | +0·061 | 9 | 0·522 | −0·006 | 0·572 | +0·047 | .. |
| Nickel | N | 36 | 1·046 | .. | 48 | 1·230 | .. | 1·151 | .. | .. |
| | SC | 73 | 1·016 | −0·030 | 31 | 1·412 | +0·182 | 1·134 | −0·017 | .. |
| | OC | 39 | 0·921 | −0·125 | 9 | 1·381 | +0·151 | 1·007 | −0·144 | .. |
| Chromium | N | 36 | 0·230 | .. | 48 | 0·178 | .. | 0·200 | .. | .. |
| | SC | 73 | 0·315 | +0·085 | 31 | 0·232 | +0·054 | 0·291 | +0·091 | 0·020 |
| | OC | 39 | 0·290 | +0·060 | 9 | 0·154 | −0·024 | 0·265 | +0·065 | 0·026 |
| Vanadium | N | 36 | 0·342 | .. | 48 | 0·455 | .. | 0·407 | .. | .. |
| | SC | 73 | 0·356 | +0·014 | 31 | 0·453 | −0·002 | 0·385 | −0·022 | 0·097 |
| | OC | 39 | 0·366 | +0·024 | 9 | 0·431 | −0·024 | 0·378 | −0·029 | .. |
| Titanium | N | 36 | 0·228 | .. | 48 | 0·212 | .. | 0·219 | .. | .. |
| | SC | 73 | 0·202 | −0·016 | 31 | 0·258 | +0·045 | 0·219 | Nil | .. |
| | OC | 39 | 0·238 | +0·010 | 9 | 0·162 | −0·051 | 0·224 | +0·005 | .. |
| Iron | N | 36 | 23·8 | .. | 43 | 20·7 | .. | 22·1 | .. | .. |
| | SC | 73 | 21·2 | −2·6 | 31 | 29·5 | +8·8 | 23·7 | +1·6 | .. |
| | OC | 39 | 24·3 | +0·5 | 9 | 21·9 | +1·2 | 23·8 | +1·7 | .. |

| | | | Over 5 p.p.m. | | | Over 5 p.p.m. | | | % over 5 p.p.m. | |
|---|---|---|---|---|---|---|---|---|---|---|
| | | No. | No. | % | No. | No. | % | Median | % | C − N |
| Lead | N | 36 | 12 | 33·3 | 48 | 25 | 52·1 | 4·75 | 44·0 | .. |
| | SC | 73 | 31 | 42·5 | 31 | 18 | 58·1 | 4·65 | 47·1 | +3·1 |
| | OC | 39 | 19 | 51·3 | 9 | 7 | 77·8 | 5·00 | 59·2 | +15·2 |

* Garden soils where a death had occurred after 10 years of more of residence at the house.
" Diff." means the difference between the mean for the cancer group (C) and that for the control (N) group. " S.E." = standard error.

where it is low. In the Devonshire locality A this might be present in the industrial waste which has found its way in the past into many of the gardens but which would only become important in regard to stomach cancer where there was also a high zinc level, and study of this problem is continuing and will be reported upon in another paper.

In the Cheshire and North Wales region, amongst the soils associated with stomach cancer those taken from ground where vegetables or fruit were being grown showed a zinc distribution somewhat different from other garden soils, as shown in Table IX, whereas no such difference appears in the control series. The organic carbon content failed to show any differences between vegetable garden and other soil. Since zinc is an active part of some enzyme systems and is

TABLE IX.—*Zinc and Cobalt Content of Soils from Vegetable or Fruit Growing Ground Compared with Other Garden Soils*

| Series | Part of garden | Zinc 0– | 20– | 50– | 80– | 140+ | Total | Cobalt 0·1– | 0·4– | 0·60– | 0·8+ | Total |
|---|---|---|---|---|---|---|---|---|---|---|---|---|
| Stomach cancer | V or F | 10 | 19 | 14 | 8 | 5 | 56 | 16 | 20 | 14 | 6 | 56 |
| | Other | 3 | 8 | 5 | 4 | 13 | 33 | 7 | 10 | 5 | 11 | 33 |
| | Exp.* | 5·9 | 11·2 | 8·2 | 4·7 | 3·0 | 33 | 9·4 | 11·8 | 8·2 | 3·6 | 33 |
| | | $\chi^2 = 14\cdot8, n = 3, P < 0\cdot01$ | | | | | | $\chi^2 = 2\cdot38, n = 2, P = 0\cdot3$ | | | | |
| Control | V or F | 12 | 16 | 9 | 3 | 6 | 46 | 16 | 16 | 9 | 5 | 46 |
| | Other | 9 | 12 | 5 | 6 | 4 | 36 | 12 | 9 | 11 | 4 | 36 |
| | Exp.* | 9·4 | 12·5 | 7·1 | 2·3 | 6·7 | 36 | 12·5 | 12·5 | 7·1 | 3·9 | 36 |
| | | $\chi^2 = 0\cdot77, n = 3, P > 0\cdot8$ | | | | | | $\chi^2 = 2\cdot45, n = 2, P = 0\cdot3$ | | | | |

* Distribution expected from that of the V–F series.

concerned with plant life and the processes of gastric digestion a connection between stomach cancer incidence and the amount present in soil is by no means wildly improbable.

Table VIII shows that in North Wales the average zinc content of 39 soils associated with other cancer is rather greater than the control figure, but for the 29 intestinal cases included the mean is 53·0, differing inappreciably from the control, and this is true also of the Cheshire cases which are all intestinal, so the excess is confined to the 7 lung cancer cases and is not statistically significant.

*Cobalt.*—In North Wales the SC soils show a range from 0·17 to 2·80 parts per million with mean value 0·655, and the N soils show a range from 0·18 to 1·04 with mean 0·522, the stomach cancer excess being twice its standard error. In Cheshire the SC range is from 0·21 to 1·55 with mean 0·625, compared with the control mean 0·528, an excess of 0·097 (t = 1·1). The combined area gives an excess of 0·121 and the Devonshire data an excess of 0·131, each of these being 2½ times their standard errors. The odds against such a result arising by chance are very great, and it must be concluded that a high cobalt level in the soil is favourable to a higher incidence of cancer of the stomach but in view of Table VIII, this seems to depend upon conjunction with some other substance, as for zinc. The other cancer series shows no significant difference from the control. Table IX shows no significant differences between the cobalt content of vegetable garden and other garden soils. The element is known, however, to be concerned in plant and animal economy and also to have carcinogenic properties, so the statistical connection between a high soil content and stomach cancer incidence is deserving of further study.

*Nickel.*—In North Wales the SC soils have contents ranging from 0·30 to 2·62 parts per million, those from Merionethshire having specially high values, and the mean of 1·016 is slightly less than that of the controls whose range is from 0·41 to 2·48. Amongst Cheshire SC soils only 29 per cent have values below 1 compared with 58 per cent in the Welsh series and the mean 1·412 exceeds the control figure by 0·182 (t = 1·6). In the Devonshire localities the nickel levels were still higher and the SC mean exceeds the N mean by 0·45 (t = 2·2). It is doubtful whether these differences indicate a connection with stomach cancer since the correlation is if anything negative in the combined Cheshire-North Wales region. Comparison between the OC and N soils shows a similar discrepancy between the two parts of this region, neither of the differences being statistically significant.

*Chromium.*—The variation in content is smaller for this element, the range in SC soils being from 0·06 to 0·92 and in the controls from 0·05 to 0·71. In North Wales the SC mean of 0·315 exceeds the control mean by 0·085 which is highly significant (t = 3·1) ; and in Cheshire there is likewise an excess of 0·054 (t = 1·5). In the combined area the stomach cancer excess is 4 times its standard error and the Devon comparison gives a similar excess of 0·096 (t = 2·8). When median chromium values based on more than one S.C. and control soils were compared in 17 separate districts, the S.C. median exceeded the control median in 11 ; but as Table X shows, the surplus incidence occurs not at the highest chromium levels but where the content lies between 0·3 and 0·6 parts per million (33 out of 89 instead of 5·5 expected).

The North Wales soils show also for the OC series, which are mostly intestinal cancer, a mean value which is 0·060 above that of the controls and in the combined area the excess of 0·065 is statistically significant. In Table X they show a concentration between 0·3 and 0·6 and for both the stomach and other cancer soils the $\chi^2$ test gives $P < 0.001$. The association with cancer seems to differ from that of zinc and cobalt in that (1) it applies to intestinal as well as to stomach cancer and (2) since chromium levels tend to be higher in *all* soils in districts where the incidence of stomach cancer is high it is not necessary to assume that chromium acts only in conjunction with some other substance.

TABLE X.—*Chromium Content of Garden Soils Directly Associated with Cancer Compared with Controls*

| Series | Stomach cancer S.M.R. | Chromium (parts per million) 0– | 0·2– | 0·3– | 0·4– | 0·6+ | Total |
|---|---|---|---|---|---|---|---|
| Non-cancer | Under 150 | 23 | 24 | 3 | .. | .. | 50 |
| | 150 and over | 19 | 8 | 1 | 1 | 3 | 32 |
| Stomach cancer | Under 150 | 13 | 6 | 11 | 6 | .. | 36 |
| | 150 and over | 14 | 17 | 10 | 6 | 6 | 53 |
| | Total | 27 | 23 | 21 | 12 | 6 | 89 |
| | Expected* | 48·0 | 30·5 | 3·8 | 1·7 | 5·0 | 89 |
| Other cancer | Under 150 | 9 | 5 | 1 | 1 | .. | 16 |
| | 150 and over | 7 | 11 | 8 | 4 | 2 | 32 |
| | Total | 16 | 16 | 9 | 5 | 2 | 48 |
| | Expected* | 26·4 | 15·6 | 2·0 | 1·0 | 3·0 | 48 |

* Expected from the control, given the same weighting according to S.M.R. of district.

*Vanadium.*—The Devonshire data show a significant excess of 0·104 (t = 2·0) when the mean vanadium content of the SC series of soils is compared with the matched control, but indications from the other areas are not clear. The means are greatly affected by occasional soils with very high amounts of the element, for example in North Wales the N series includes one with 6·20 p.p.m., the next highest value being 0·80, and in Cheshire N soils the highest values were 3·44 followed by 0·97. Table XI, which avoids this difficulty, reveals a great difference between the distributions of N soils in the groups of districts with high and low stomach cancer mortality, two thirds of the soils in the former group having less than 0·2 p.p.m. compared with one eighth in the latter. When the SC soils are compared with the distribution expected from controls within the same district groups the difference is hardly significant ($\chi^2$ = 9·1, n = 4, P = 0·06), and a difference of the same kind is seen for other cancer, namely an excess of soils with content around 0·3 parts per million. No definite conclusions can be drawn, but there is a curious resemblance to the chromium comparisons in Table X.

TABLE XI.—*Vanadium Content of Garden Soils Directly Associated with Cancer, Compared with Controls*

| Series | Stomach cancer S.M.R. | Vanadium (parts per million) 0– | 0·2– | 0·4– | 0·6– | 0·8+ | Total |
|---|---|---|---|---|---|---|---|
| Non-cancer | Under 150 | 6 | 15 | 19 | 6 | 4 | 50 |
| | 150 and over | 21 | 6 | 2 | 1 | 2 | 32 |
| Stomach cancer | Under 150 | 7 | 14 | 5 | 6 | 4 | 36 |
| | 150 and over | 26 | 15 | 5 | 2 | 5 | 53 |
| | Total | 33 | 29 | 10 | 8 | 9 | 89 |
| | Expected* | 39·1 | 20·7 | 17·0 | 6·0 | 6·2 | 89 |
| Other cancer | Under 150 | 4 | 6 | 4 | — | 2 | 16 |
| | 150 and over | 11 | 14 | 2 | 1 | 4 | 32 |
| | Total | 15 | 20 | 6 | 1 | 6 | 48 |
| | Expected* | 22·9 | 10·8 | 8·1 | 2·9 | 3·3 | 48 |

* Expected from the control, given the same weighting according to S.M.R. of district.

*Titanium.*—The analyses relate to the titanium extractable by the standard solvent used in preparing the soil for spectrographic study, the insoluble forms such as rutile which are plentiful in soil not being thought likely to have any biological activity. In none of the areas is there any indication of any connection with cancer.

*Iron.*—In Devonshire and North Wales the stomach cancer soils do not differ significantly in average content from the controls, the mean levels being if anything below expectation. In Cheshire, however, the SC series shows an excess of 8·8 parts per million (t = 3·0). No appreciable differences appear for other cancer.

*Lead.*—Since the quantitative assessment of amounts of this element by the spectrographic method is difficult when the level exceeds 5 parts per million, the statistical comparison has been made by comparing proportions of the total soils having 5 or more p.p.m., and by the median values, these measures being unaffected by uncertainties as to the exact values in the upper part of the scale. The Devonshire data show a lower median for the stomach cancer series than for the controls, and 23 per cent of each series had 5 or more parts per million of lead. In the Cheshire-North Wales area there was no significant difference by either measure, but soils connected with non-gastric cancer show greater proportions with a high lead content than the controls, this series consisting mainly of intestinal cancers.

*Copper.*—The amounts of copper in the soils from the two Devonshire districts were determined by a colorimetric process in a separate acetic acid extract of the soil, as described below. Table VI(b) shows that the mean content of the soils from houses where stomach cancer had occurred is higher by 0·50 p.p.m. than that of the controls. The ratios of cobalt to copper are about 0·18 in each group, and the ratios of nickel to copper are 0·7 in each group, giving no indication that copper has a counteracting effect. The ratio of zinc to copper, however, is 6·8 in the stomach cancer soils compared with 4·7 in the controls.

### SUMMARY

Chemical and spectrographic study of garden soils in North Wales, Cheshire and two localities in Devonshire has established correlations between the amounts of certain constituents and the frequency of cancer of the stomach. Organic matter, zinc and cobalt are related positively and significantly with stomach cancer incidence but not with intestinal cancer, whilst chromium is connected with the incidence of each of these. The abnormal rates of stomach cancer in parts of North Wales are associated with long residence on soils whose organic content lies between definite limits. Soil rich in zinc or cobalt is found with excessive frequency where a case of stomach cancer has occurred but the geographical distribution of such soils appears to be unrelated to that of stomach cancer rates. Vanadium and iron show inconclusive relations with stomach cancer in one of the areas, whilst nickel, titanium and lead show no connection anywhere with this form of cancer.

### METHODS OF TRACE ELEMENT ANALYSIS

Soils were examined for elements likely to be taken up by plants in micro- or trace quantity. The likelihood of this uptake is to some degree related not to the total quantity of each element in the soil, but rather to a combination of quantity weatherability and ease of solubility. To simulate this " availability for plants " it is customary to extract soils with very dilute acetic acid or neutral salt solutions or even with dilute solutions of ion complexing agents. For the present investigation N/2 acetic acid was chosen (20 g. soil/800 ml. acid) the acid being in contact with the soil with occasional shaking for 12 hours. Analysis of the acetic acid extract then followed closely the procedure of Mitchell (1945). This involves a separation of the micro-elements from those present in large amounts (e.g. K, Ca, Na, Mg) resulting in a concentrate of the micro-elements. The precipitate of all these elements is ultimately arced by direct current, the arc light being examined by a Hilger Large Quartz Spectrograph.

In the present investigation 30 mg. $Al_2O_3$, 2·5 mg. $Fe_2O_3$ and 0·4 mg. Cd. were introduced before precipitation to ensure consistent arcing and to provide elements for reference in the spectrogram. Cobalt, Nickel, Chromium, Vanadium, Titanium, Lead and Zinc were then determined quantitatively on the spectrogram using Iron, already found by chemical analysis, as a reference standard. Zinc was also determined by using the added Cadmium as reference, and it is this second value which has been used throughout the present work.

Available copper cannot be determined spectrographically because of limitations imposed by contamination. It was necessary therefore to devise a separate method, an adaptation of the colorimetric estimation by Zinc Dibenzyldithiocarbamate (Andrus, 1955). For this a fresh extract was prepared (20 g. soil by 800 ml. N/2 acetic acid). The extract was evaporated to dryness, oxidized with a little nitric acid and dissolved in N. acetic acid. This was shaken with a solution of Zince Dibenzyldithiocarbamate in carbon tetrachloride and the intensity of colour produced in the latter by copper was estimated with a spectrophotometer.

We are greatly indebted to the Medical Officers of Health and their Assistants in Cheshire, the 5 counties of North Wales and the 2 districts of Devonshire for the collecting of soil samples and for supplying the information as to deaths from cancer and other causes. Our thanks are due also to the British Empire Cancer Campaign for supporting the work and to the Analysts and Technicians in the Department of Agricultural Chemistry at Bangor whose services have been provided by the Campaign.

### REFERENCES

ANDRUS, S.—(1955) *Analyst*, **80**, 514.
DAVIES, R. I. AND WYNNE GRIFFITH, G.—(1954) *Brit. J. Cancer*, **8**, 56, 594.
HUXLEY, J.—(1958) ' Biological Aspects of Cancer ', London (Allen & Unwin).
MITCHELL, R. L.—(1945) ' Spectrographic Analysis of Soils, Plants and related Materials ', Technical Communication 44. Harpenden (Commonwealth Bureau of Soil Science).
STOCKS, P.—(1955) *Rep. Brit. Emp. Cancer Campgn*, **33**, 468.—(1956) *Ibid.*, **34**, 520.—(1958*a*) *Ibid.*, **36**, 342.—(1957) *Ibid.*, **35**, Supplement, p. 95. —(1958*b*) In ' Cancer ', London, (Butterworth), Chapter 4, Vol. 3, p. 153.
WALKLEY, A. AND BLACK, I. A.—(1934) *Soil Sci.*, **37**, 29.